# Ultrasound Transmission Tomography for Detecting and Measuring Cylindrical Objects Embedded in Concrete

**DOI:** 10.3390/s17051085

**Published:** 2017-05-10

**Authors:** Dalmay Lluveras Núñez, Miguel Ángel Molero-Armenta, Miguel Ángel García Izquierdo, Margarita González Hernández, José Javier Anaya Velayos

**Affiliations:** 1 Instituto de Tecnologías Físicas y de la Información, ITEFI (CSIC), C/Serrano 144, 28006 Madrid, Spain; d.lluveras@csic.es (D.L.N.); jj.anaya@csic.es (J.J.A.V.); 2 Innerspec Technologies Europe, Av. Madrid km. 27.2, 28802 Alcalá de Henares, Spain; mmolero@innerspec.com; 3 E.T.S.I. Telecomunicación, U.P.M. Av. Complutense 30, 28040 Madrid, Spain; miguelangel.garcia.izquierdo@upm.es

**Keywords:** ultrasound, tomography, concrete, inspection system

## Abstract

This study explores the feasibility of using transmission tomographic images based on attenuation measures in transmission to detect and estimate the most common materials that are embedded in concrete, reinforcements and natural and artificial voids. A limited set of concrete specimens have been made in which cylindrical objects such as bars/tubes of steel, PVC and aluminium have been embedded to analyse the effect of size and material. The methodology and scope of this study is presented and numerical simulations are carried out to optimize the emitter-receiver configuration and to understand the complex physical propagation phenomena of ultrasonic signals that travel through concrete with embedded inclusions. Experimental tomographic images are obtained by using an ultrasonic tomographic system, which has the advantage of needing only two ultrasonic transducers. Both the software simulation tool and the tomographic inspection system are developed by the authors. The obtained results show that PVC tubes and steel bars of diameters higher than 19 mm and embedded in cylindrical specimens, can be detected and their sizes estimated using segmented tomographic images.

## 1. Introduction

The use of ultrasonic images to evaluate the quality and degradation state of concrete structures by the identification of heterogeneities, defects or cracks is an attractive solution to the diagnosis of civil infrastructures [1,2]. In the next years, large investments for the rehabilitation of these infrastructures are expected both in Europe as America [3,4], and non-destructive techniques (NDT), are an attractive solution that allows reducing rehabilitation costs among others [3]. Problems such as microcracking, voids, reinforcement and tendons deteriorated, require detection by using NDTs and are well suited for evaluation with the use of ultrasonic imaging [2,5].

In recent years, ultrasonic imaging, and tomographic imaging in particular, has gained increasing importance in the evaluation of cementitious materials by using different techniques, equipment, frequencies, and wave types [6,7]; especially for detecting and locating holes, ducts, cracks, and thickness measurements, [8,9,10]. Tomographic images allow the visualization of the cross section of structures, thus enabling better detection of defects, cracks, or discontinuities in the material. Several authors have studied the propagation considering the diffraction effect by using straight rays [11]; others have investigated the attenuation tomography [12] or the tomography by surface waves [13]. Haach and Juliani [14] presented a study of tomography reconstruction based on the results of ultrasonic tests in concrete prisms with distinct compositions.

A tomographic image is obtained from projections through the object. These projections consist of velocity or attenuation measurements of ultrasonic pulses transmitted through the specimen. The most popular methods of tomographic reconstruction are the algebraic techniques, such as the algebraic reconstruction technique (ART) [15] and the simultaneous iteration technique (SIRT), which are based on the resolution of the system of linear equations generated by the projections [16].

The tomographic images were obtained by an immersion ultrasonic tomography system that operates in transmission mode [17], which has the advantage of needing only two ultrasonic sensors. This system allows inspections in situ of specimens or cores. Several concrete specimens with embedded bars or tubes of different sizes and materials are inspected. To optimize the emitter-receiver configuration and select the optimal parameters for the inspection, several simulations are carried out with the software SimNDT [18,19].

## 2. Methodology

The objective of the work is to evaluate if ultrasonic transmission tomographic images based on attenuation measures can locate and estimate the most common materials that are embedded in concrete, reinforcements and natural and artificial voids. To achieve this aim end, a limited set of concrete specimens have been designed and manufactured to study of the most relevant parameters.

In this paper we refer to the cylindrical objects embedded in concrete as inclusions. In order to study the behaviour of the most common inclusions found in concrete, i.e., reinforcements and voids, cylindrical objects such as steel bars and PVC tubes of three different diameters have been embedded. These two materials yield high and low acoustical impedance (steel ≈ 46 × 10^6^ Pa·s/m, air ≈ 0 Pa·s/m) compared to concrete (≈10 × 10^6^ Pa·s/m). In order to cover a complete range of acoustical impedance, aluminium embedded inclusions (≈17 × 10^6^ Pa·s/m) have been added.

The diameters of the inclusions are similar to the wavelength of the ultrasonic transducers and are near to the detectability threshold. The design of specimens allows us to vary the number and size of the inclusions in the different tomographic images.

Concrete is a heterogeneous material. Therefore, ultrasonic waves have a complex propagation pattern in which different physical phenomena, such as reflection, diffraction, scattering and mode conversion interact in such a way that can mask the relevant information about embedded structures [20]. In order to consider this aspect, a high strength concrete has been used in which the maximum size of the aggregates is similar to that of the embedded inclusions. Ultrasonic images of a highly heterogeneous concrete with multiple inclusions are complex to interpret. Therefore, detection and estimation of embedded materials are difficult problems [2].

The first step to evaluate the attenuation tomographic image as a valid tool for locating and estimating the embedded inclusions in concrete has been done by numerical simulation. Description of the simulations, their parameters and the obtained results are found in Section 4.

After the simulation study, the experimental validation has been carried out. Concrete specimens have been made in which the inclusions have been embedded. An ultrasonic tomographic system has also been designed and constructed by the authors, allowing the collection of all necessary data for a complete inspection (due to the massive amount of data required for the complete inspection of the specimens, collection of these is not feasible by hand). The complete specifications of the specimens and the ultrasonic system are found in Section 5. Section 6 shows several intermediate results of the inspections, like B-scans, sinograms, reconstructed images and finally the detection and estimation of the sizes and positions of the embedded inclusions.

Next section, as a previous step to the presented methodology, shows the mathematical bases of the algebraic reconstruction methods that allow obtaining the ultrasonic attenuation tomographic images from the collected data.

## 3. Algebraic Reconstruction Algorithms

The attenuation of the *m*th ray, $p_{m}$, (1 < *m < M*) of an acoustic wave that travels through the path l, between two points (from the emitter to the receiver) can be expressed by the following path integral:

(1) $$p_{m}=\underset{l}{\int }\alpha \left(l\right)dl$$

where α(l) is the attenuation spatial-distribution.

**Figure 1:**
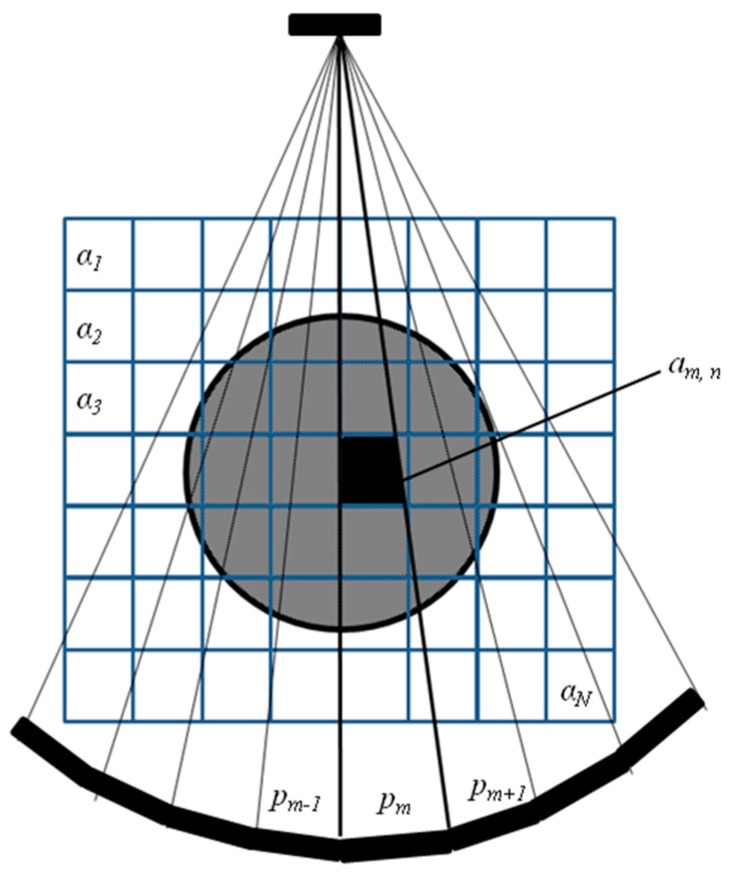
Fan tomographic inspection that considers the discretization of the attenuation spatial-distribution.

Algebraic tomographic reconstruction techniques are based on the discretization of the former spatial-distribution with an N cells grid, as shown in Figure 1. Thus, the expression in Equation (1) takes the following form:

(2) $$p_{m}=\overset{N}{\underset{n=1}{\sum }}a_{m,n}\;\alpha_{n}$$

where $\alpha_{n}$ is the attenuation that produces the *n*th cell, *1* < *n* < *N*, and $a_{m,n}$, is the ray area in each of the cells that the path passes through. Here, it is interesting to note that most of the $a_{m,n}$ coefficients are zero because the path does not pass through them. The result of a complete inspection is a sinogram, which gathers all the attenuations into a unique image.

A tomographic inspection usually consists of thousands of these equations (rays), which form a system of linear equations that, for convenience, is expressed here with vector algebra:

(3) p=Aα

where $p={\left[p_{1},p_{2},\ldots ,p_{M}\right]}^{T}$ is the vector of measured attenuations, $\alpha ={\left[\alpha_{1},\alpha_{2},\ldots ,\alpha_{N}\right]}^{T}$ is the vector that contains the discretized values of the attenuation spatial-distribution, and ***A*** is an M × N matrix with generic element *a_m,n_*, which is the area of the *m*th ray, *1 < m < M*, into the *n*th cell, *1 < n < N*. Thus, the tomographic reconstruction consists in determining the vector *α* as:

(4) $$\alpha =A^{-1}p$$

This solution implies the inversion of the *A* matrix. However, this is an ill-conditioned and disperse matrix (many rows are linearly dependent and most of the row elements are zero). Thus, solving this system by applying standard procedures, such as direct matrix inversion, least squares, or singular value decomposition, is not possible. Most of the techniques for solving Equation (4) are based on specific iterative methods.

Algebraic reconstruction methods update the solution by successively processing each equation (ray) separately. Beginning with an initial solution, ***α***^(0)^, which is projected on the hyperplane represented by the first equation in the system of equations (Equation (4)), ***α***^(1)^ is obtained, which in turn is projected by the hyperplane represented by the second equation to yield ***α***^(2)^, and so on. The process can be mathematically described by:

(5) $$\alpha^{\left(k+1\right)}=\alpha^{\left(k\right)}+\lambda \frac{p_{\left(k+1\right)}-a_{\left(k+1\right)}^{T}\alpha^{k}}{{\left\|a_{\left(k+1\right)}\right\|}^{2}}a_{\left(k+1\right)}$$

where $a_{\left(k+1\right)}^{T}$ is the (*k* + 1)*th* row of the matrix ***A***. The relaxation parameter is denoted as *λ*, which varies between 0 and 1; the smaller this factor is, the more time-consuming the solution process, but the better the quality of the solution.

The SIRT algorithm uses Equation (5) but modifies the solution, considering at each iteration the effect of all ray paths crossing each cell. By applying the SIRT algorithm, better solutions are usually obtained at the expense of a lower convergence speed. This is the algorithm used in the present work.

## 4. Numerical Simulations

Different simulations have been done to evaluate the propagation of ultrasonic waves in concrete, considering it as a heterogeneous medium, as well as with the presence of inclusions. Ultrasonic tomography inspection in highly heterogeneous materials such as concrete requires studying a complex wave propagation consisting of reflection, dispersion, scattering, and mode conversion, which can mask relevant information regarding the material structural properties. For this purpose, the simulation software SimNDT, developed by the authors [18,19], was used to generate two-dimensional (2D) numerical simulations.

**Figure 2:**
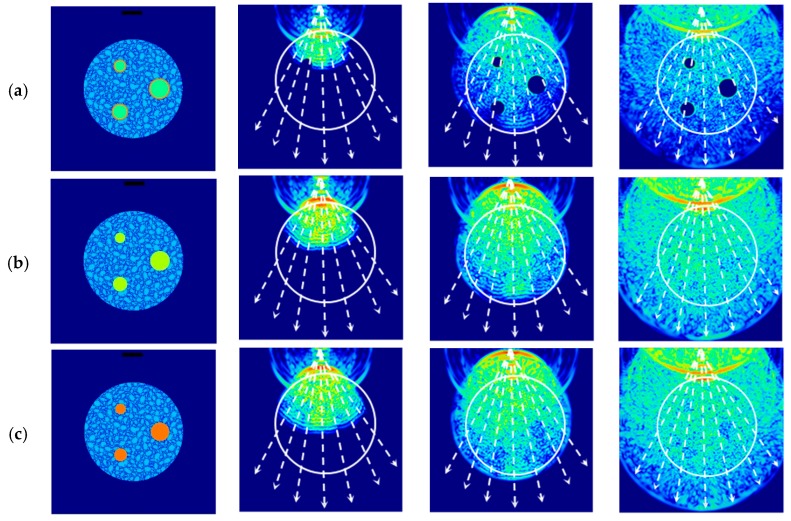
Scenarios and snapshots of the propagation of ultrasonic waves in concrete with: (**a**) PVC inclusions; (**b**) aluminium inclusions; (**c**) steel inclusions.

The same scenarios and materials were used in the numerical simulations and the experiments. The designed scenarios were 2D models, as shown in Figure 2 (column 1). These represent a 250 mm square water medium with a circular specimen (Ø 150 mm) and three circular inclusions of different diameters, as shown in Table 1. The selected inclusion materials were PVC, aluminium and steel.

The concrete scenario was modelled as a three-phase material formed by a cement matrix, aggregates (60% and max. size of 20 mm), and air voids (2%). Table 2 shows the material properties used for the numerical simulations software [19].

The inspection setup consisted of a ring (Ø 240 mm) of 119 transducers (Ø 25 mm), equally angular spaced at 3° and operating in transmission mode. The longitudinal wave was simulated with a 500 kHz four-cycle Gaussian envelope pulse (wavelength in concrete ≈ 8 mm).

Figure 2 presents the scenarios and three consecutive snapshots of the wave propagation through the concrete with circular inclusions. The trajectories of the rays (white dotted lines) can be received by the transducers for a transmitter placed at the middle of the top edge. The ultrasonic wave propagates uniformly in water; however, when the wave reaches the concrete and its inclusions, reflection, diffraction, and scattering take place. As a result, the wavefront is modified, and the inclusions cause a variation in the amplitude of the wave, which is dependent on the wave properties and geometry. Because the diameter of the transducer is similar to that of the inclusions, the wavefront may be slightly affected.

Figure 2a (concrete with embedded PVC inclusions) shows that when the differences in acoustic impedance between the concrete and the inclusions are high, the wavefront does not travel through the inclusions. On the other hand, Figure 2b (concrete with embedded aluminium inclusions) shows that when the acoustic impedances of the aggregates and the aluminium are similar, as shown in Table 1, the wavefront travels through the inclusions. Finally, Figure 2c (concrete with embedded steel inclusions) shows that when the acoustic impedance of the steel is higher than that of the aggregates, the wavefront is attenuated when it travels through the inclusion and the resulting image contains a low contrast shadow of the inclusions. The effectiveness of the inclusion detection is affected by the reflection/transmission produced in the concrete/inclusion/concrete interface and by the diameter-wavelength relation, similarly to the conclusions reported in [6].

## 5. Experimental Design

The experiments studied the behaviour of the wave propagation and the tomographic reconstruction in standard concrete specimens to obtain information about the positions and sizes of the bars or tubes.

### 5.1. Ultrasonic Tomographic System

**Figure 3:**
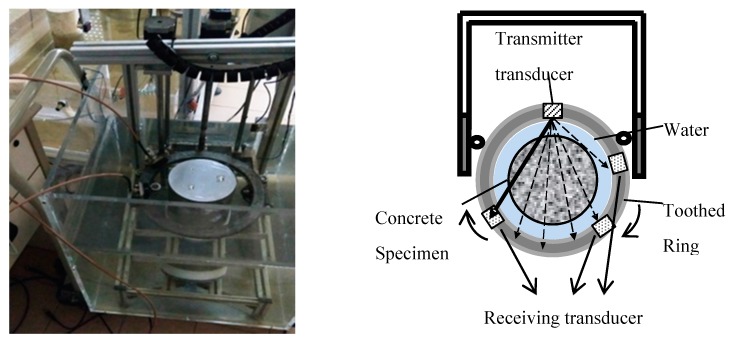
Portable inspection system for immersion ultrasonic tomography.

A portable inspection system for immersion ultrasonic tomography was developed by the authors [17]. The system, as shown in Figure 3, is easy to transport and allows in situ inspections of specimens or cores, as well as ultrasonic inspections in transmission for specimens with cylindrical symmetry so as to produce an image. This system could inspect specimens up to 150 mm of diameter but could be scalable. The system consists of two subsystems: one for inspection, and the other for generation of ultrasonic images.

The inspection subsystem is composed of a small portable tank (400 × 400 × 500 mm^3^), a mechanical scanner, and an electronic control. The mechanical scanner is resistant to water immersion and has three different motions: a rotation movement centred on the axis of the specimen cylinder, a vertical movement along the specimen height, and a rotation of the receiving transducer over the toothed ring, as shown in Figure 3. The toothed ring structure guides the movement of the transducers and varies the relative position between the emitter and the receiver; this aluminium ring has an internal diameter of 240 mm and an external diameter of 270 mm. The movements of this system have the advantage of needing only two transducers to generate many emitter-receiver positions for tomographic scanning, as shown in Figure 3 (dotted line).

The electronic control has two functions: to arrange the movements of the transducers with respect to the specimen and to produce a synchronism signal associated with the relative position of the transducers in the specimen. The electronic system is designed to operate in situ with 24 V and consuming 60 W. The immersion in water favours the propagation of ultrasonic waves and maximizes both the emitted energy and the extraction of characteristics from the received signals.

Sendas equipment (CSIC, Madrid, Spain) is used for the emission, amplification, and reception of the signals [21]. Two v413 500 kHz wideband transducers (Panametrics, Waltham, MA, USA) are used. A 1 µs and 400 V rectangular pulse is applied as excitation signal, and a variable gain receiver amplifier of between 20 and 60 dB is used. The sampling frequency is 10 MHz.

**Figure 4:**
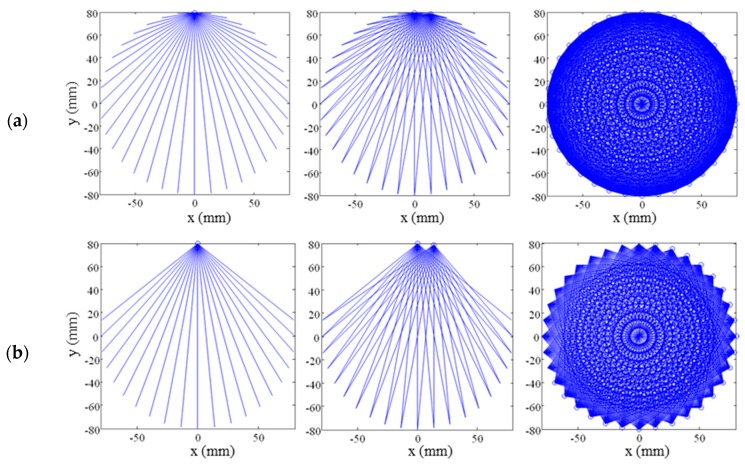
Simulation of rays for the tomographic scan: (**a**) without the restriction angle and (**b**) with a restricted angle of 75°.

The system parameters for the tomographic inspection imply the setting of the number of transmitters, the number of receivers, the restricted angle, and the diameter of the transducer ring. The restricted angle is the minimum angle between the emitter and the receiver transducer, defined to eliminate the received information in the receivers closest to the transmitter, as shown in Figure 4. These signals could be due to reflection or conversion of the propagation modes; they do not improve the information about the specimen.

### 5.2. Concrete Specimens

**Figure 5:**
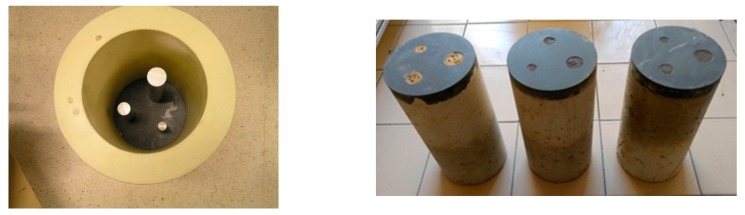

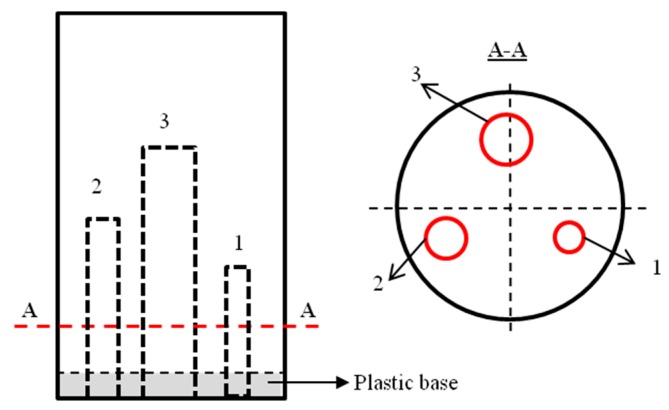
Evaluated cylindrical concrete specimens.

Concrete specimens with different sizes and inclusion materials (aluminium, steel, and PVC tubes) were made. Three inclusions of different diameters and heights, as shown in Table 3, were placed in each mold before fabrication at different heights (Figure 5). The bars and tubes were fixed in a plastic base that was placed into the mould before the specimen fabrication, since the specimen height was 270 mm. The PVC tubes were sealed to prevent their filling with concrete during the manufacturing process.

Three cylindrical specimens (Ø 150 mm × 300 mm) were moulded according to the EN 12390-2 norm [22] and kept in a laboratory environment for 1 day. The specimens were then removed from the mold and stored in water over a 28 day curing process until the inspections were carried out.

The materials used to manufacture the concrete specimens were Portland white cement type CEM I 52.5R, silica sand (max. size: 4 mm), crushed limestone aggregates (max. size: 30 mm), and ViscoCrete 5980 superplasticizers to improve workability. Table 4 shows the concrete mix proportion used in this experiment.

### 5.3. Inspection Procedure

The procedure used to generate tomographic images consists of three movements. The first movement is a vertical displacement along the height of the specimen, carried out with a step 2 mm in height. The second movement establishes the relative position between the emitter and the receiver. In this case, the inspection angle is 220°; accordingly, the restriction angle is 70°. The last movement is the rotation of the specimen, which is equivalent to the motion of the emitter transducer. The programmed movement is equivalent to having 100 emission positions. Therefore, each revolution of the specimen generates A-scans coming from 100 emitter positions times 220 receiver positions, resulting in 100 B-scan images of 220 signals taken at 2 mm each, or 22,000 A-scans. The diameter between the transducers is 242 mm.

## 6. Results and Discussion

**Figure 6:**
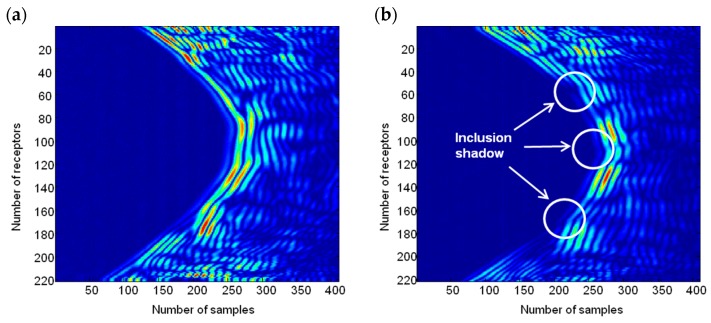
B-scan of the concrete specimen: (**a**) without inclusions; (**b**) with 3 with PVC tubes.

Figure 6 shows a B-scan image of the concrete specimen with PVC tubes at a height without PVC tubes (Figure 6a) and at a height with three PVC tubes (Figure 6b). The images show the set of signals collected by the 220 receivers. The wavefront is curved due to the relative positions between the emitters and the receivers. The presence of inclusions causes small footprints in the wavefront (Figure 6b).

**Figure 7:**
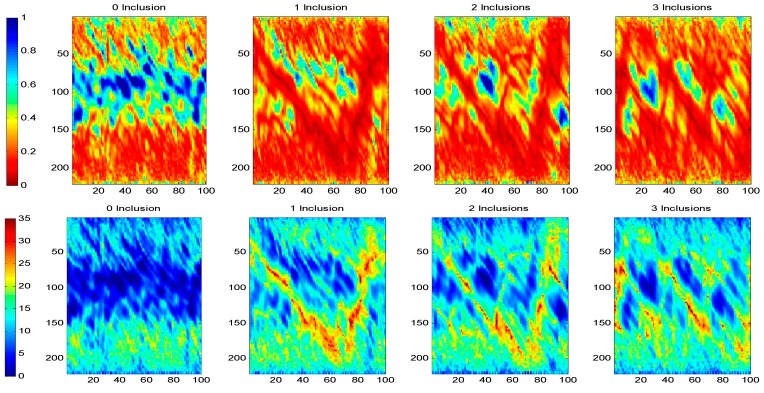
Comparison between lineal and logarithmic sinograms.

Figure 7 shows a comparison between the linear and the logarithmic amplitude sinograms for the concrete with PVC tubes specimen as well as the tomographic reconstruction. Each point of the sinogram is computed by using the maximum value of the first semi-pulse form of the received ultrasonic signal corresponding to each A-scan. In the figures, the *y*-axis represents the receiver positions, and the *x*-axis denotes the emitter positions. As the number of PVC tubes increases, it becomes more difficult to determine the different contributions of each tube to the sinogram, due mainly to the heterogeneous behaviour of concrete.

By applying logarithmic transformation, it is possible to reduce the dynamic range of the amplitude of the received ultrasonic signal. In this way, the attenuation produced by the inserted tubes in the sinogram is highlighted (red colour). From here on, only logarithmic sinograms and their reconstructed tomographic images are presented in this paper.

**Figure 8:**
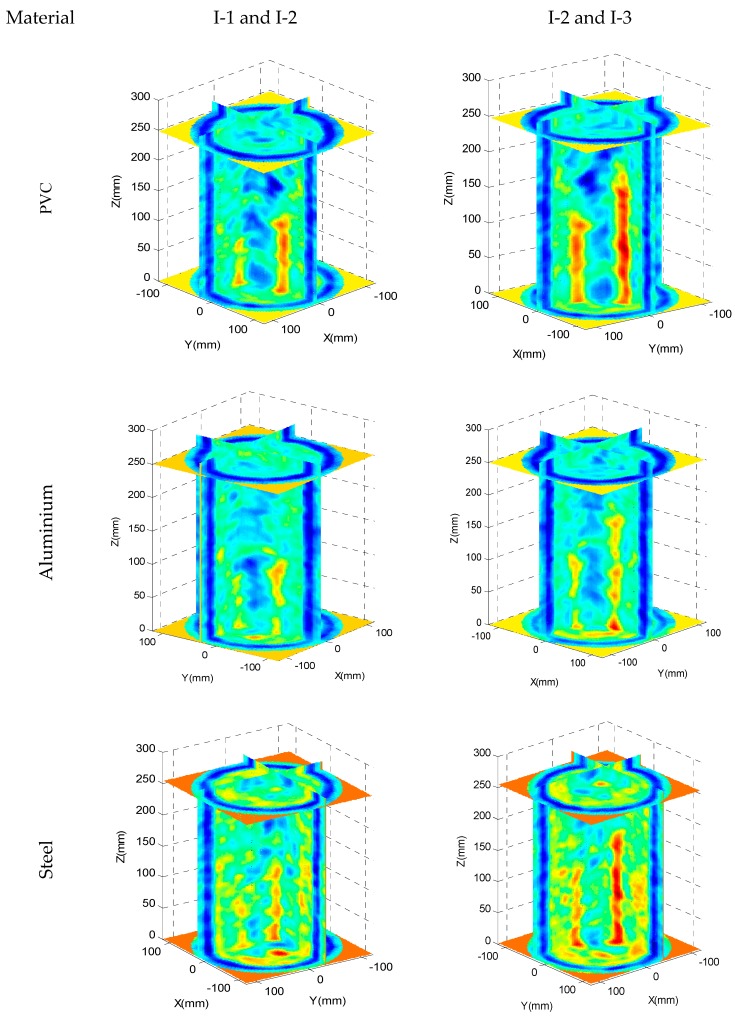
3D representation of the tomographic reconstruction.

In Figure 8 a 3D representation of the tomographic reconstruction is presented for each concrete specimen (two for each one). Two vertical planes including the positions of the embedded bars/tubes are shown. The results obtained agree with the simulations performed. When the difference in acoustic impedance is high, for example concrete/air and concrete/steel, the two inclusions of greater section (I-2 and I-3) are well delimited in position and size. However, the embedded inclusion I-1 is not clearly visible in all its length. When the acoustic impedances are very similar, concrete/aluminium, the bars is not delimited in all its extension. High attenuation zones can also be seen, which do not correspond to the position of the bars. These zones are, often on the edges of the test probes, and are due to concrete natural voids. It is necessary to take into account that the maximum size of the aggregates used is 30 mm so the dispersion caused by them provokes the difficulty of detecting aluminium and steel bars.

**Figure 9:**
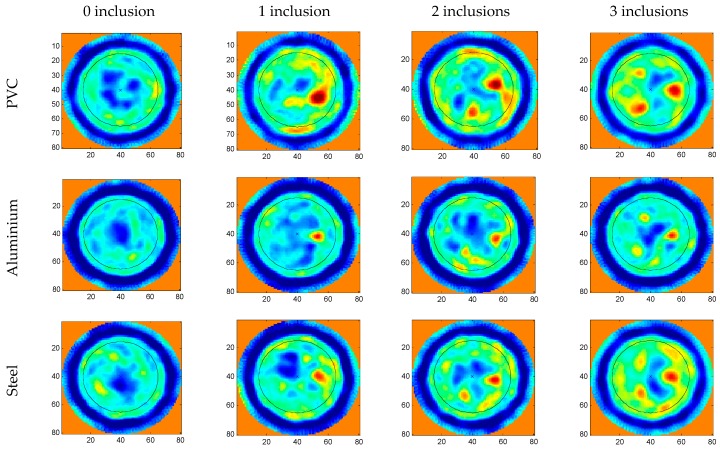
Experimental tomographic reconstruction images of the concrete specimens.

A series of tomogram slices have been selected where 0, 1, 2 and 3 inclusions are detected in order to show partial results, Figure 9. All the images are 80 × 80 pixels; each side of an image is physically equivalent to the external diameter of the ring of the inspection system, which means that each pixel represents a 3 mm length. Thus, the concrete specimens, as shown in the reconstructed tomographic images, are 50 pixels in diameter. A blue external ring can also be observed in the experimental reconstruction images. This ring is formed by the multiple echoes produced in the aluminium ring of the inspection system.

All the tomographic images show the specimen sizes. The bar/tube positions and relative sizes are obtained quite accurately regardless of the number of bars/tubes. The bars/tubes inserted in the concrete modify the ultrasonic image in different ways depending on the material; in all cases, it is possible to correctly identify the different inclusions from the reconstructed tomographic images.

At the centre of all the specimens is a high-amplitude zone (low attenuation). When the transducers are not diametrically opposed, (i.e., when the path between the emitter and the receiver does not cross the central zone of the test probes), the received signal is highly attenuated even though the path that the ultrasonic signal travels through the specimen is shorter than in the diametrically opposed case. This effect is partially corrected by an apodization procedure, implemented through the inspection system by applying different gains as a function of the emitter-receiver positions, (i.e., low gains when diametrically opposed, within the limits of the acquisition dynamic range).

In all the images, the water-specimen interface (blue zones on the edges of the test specimens) is hardly visible because the transmitted pulse is often hidden by the multiple echoes of the aluminium ring of the ultrasonic tomographic system. Other high attenuation zones are also observed corresponding to natural voids in concrete which are normally located near the surface of the specimen.

To evaluate the quality of the ultrasonic reconstructed images, a segmented image based on a threshold of the image maximum is generated. The size assigned to the defect depends on the segmentation threshold. In this case, the criterion applied requires that the reconstructed images of steel bars, with intermediate attenuation, are fitted to the real test probes. In NDT-US, the threshold for detecting a defect or inclusion is determined using a standard specimen, for example, a bore of known diameter on a specimen of the same material to be inspected [23]. In our case, we have used the iron bar with greater thickness as inclusion pattern. This threshold has been applied to all ultrasonic images in order to obtain an estimate of the size of the different inclusions. As in other research areas where ultrasonic imaging is used (welding, aeronautical material, medicine, etc.), it would be necessary to manufacture a specimen with the same characteristics that the structure to be inspected: concrete type, reinforcement, dosage, etc. Therefore the characteristics and the state of the inspected structure would be analyzed in comparison with the image of the specimen that has been used to calibrate the tomographic system.

**Figure 10:**
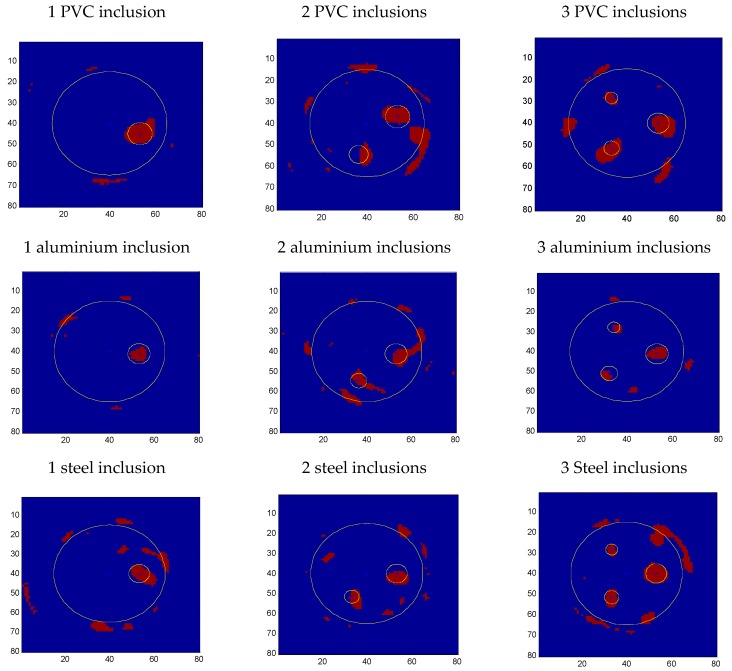
Segmented images of the tomographic reconstructions.

A 30% threshold has been applied in different sections of the selected images, see Figure 10. In the images with only one inclusion appears the largest diameter inclusion, (I-3), in the images with two inclusions appear the two largest diameters inclusions (I-3 and I-2) and in the images with three inclusions the three inclusions appear (I-3, I-2 and I-1).

The processed image (80 × 80 pixels) in Figure 10 shows the differences between the real and the estimated dimensions of the inclusions. In the case of the PVC tubes, the estimated size is similar to the real dimensions of the inclusions, which are slightly oversized. In the case of the aluminium bars, the estimated size is smaller than the real dimensions of the test probe bars. This effect can be explained by the simulation presented in Section 3: the aluminium bars, having an acoustic impedance close to that of concrete, slightly diminish the amplitude of the travelling ultrasonic signal.

The positions of the inclusions are obtained with good accuracy, but with a slight displacement by a few millimetres toward the edge of the test pieces. This effect is due to the segmentation threshold, which is sensitive to the non-diametrically opposed positions of the transducers. Most of the inclusions have ellipsoid shapes, with the major axis radially oriented due to the above-mentioned reason.

To evaluate the quality of the estimations two objective measures related with the area and the diameter of the inclusions have been defined from the segmented images for all tomogram slices.

The estimated area ($\hat{A}$ (mm^2^), has been obtained from the number of connected pixels near to the inclusion position and considering that a pixel area is 9 mm^2^. The area estimation error, $\varepsilon_{A}$ (%), has been defined as:

(6) $$\varepsilon_{A}\left(\%\right)=\frac{\left(\hat{A}-A\right)}{A}\cdot \;100$$

where, *A* (in mm^2^) is the area of the real inclusion.

The mean diameter of each inclusion ($\bar{D}$ in mm) has been calculated from the estimated area as:

(7) $$\bar{D}=2\cdot \;\sqrt{\frac{\hat{A}}{\pi }}$$

The diameter estimation error of the inclusions, $\varepsilon_{D}$, has been calculated as:

(8) $$\varepsilon_{D}\left(mm\right)=\bar{D}-D$$

where, *D* is the diameter of the real inclusion. It should be noticed that the diameter error is in millimetres and the pixel length is 3 mm. 

The real and estimated dimensions, area and diameter, of bars and tubes embedded in concrete specimens are shown in Table 5, as well as the error and the standard deviation. In this table is shown the mean of all areas and diameters of the detected bars/tubes. The column called detectability (%) indicates the percentage of tomograms where the inclusions are detected, considering that it is detected if $\hat{A}$ > 0.1 × *A*. The two upper tomograms of each bar have also been considered as not detectable to eliminate the edge effect in the estimations

In Figure 8 it can be seen that not all the tomograms detect the embedded materials. Detectability of steel bars and PVC tubes are similar, both have different acoustical impedances compared to concrete resulting in high contrast reconstructed images. On the other hand, aluminium bars with a similar acoustical impedance to concrete have the lowest detectability figures. Regarding the sizes of the inclusions, I-1 inclusions are difficult to detect because their size is similar to the size of concrete aggregates and the ultrasonic wavelength. Again, steel bars and PVC inclusions behave in a similar way while aluminium bars have the lowest figures. These results agree with the numerical simulation conclusions.

From the tomograms that detect the inclusions, dimensions have been estimated. The steel bars with larger diameter (I-3) have been used to define the threshold for the segmented images. By this reason, their error is zero. The estimation error in the steel bar I2 is good that indicates that the selected threshold is correct to estimate the diameter of steel bars PVC tubes are overestimated while aluminium bars are underestimated but in all cases the absolute errors are lower or equal than 4 mm. The detectability of aluminium bar is less with the others steel bars and PVC tubes, due to the differences in the acoustic impedance and elastic properties. Similar results were obtained by [24].

## 7. Conclusions

This study explores the feasibility of using attenuation tomographic images based on attenuation measures to detect and estimate the most common materials that are embedded in concrete, reinforcements and natural and artificial voids. A limited set of concrete specimens have been made in which bars/tubes of steel, PVC and aluminium have been embedded to analyse the effect of size, material and multiplicity of the inclusions.

An automated immersion ultrasonic inspection system for cylindrical specimens was used to obtain the experimental data. This system allows the detection of heterogeneities, such as voids, inclusions, and discontinuities, in materials. As a previous step to the experimental data capture process, several simulations were carried out for two main reasons: to obtain a much better understanding of the complex scattering process in concrete and to select the parameters of the ultrasonic tomography inspection system.

The results showed that the inclusions, regardless of the material, produce a high attenuation zone on the reconstructed tomographic images, but with only attenuation tomographic images is not possible to determine the type of embedded material. The positions and diameter of the inclusions were estimated from the segmented images. The precision of these measurements depend on the acoustical impedance relation between the concrete and the embedded material and their diameter. Multiple inclusions with a minimum diameter higher than of two wavelengths (approximately 16 mm in concrete) could be detected. The positions of all bars/tubes were detected. The sizes of PVC and aluminium inclusions were oversize and undersize, respectively. The effectiveness of the inclusion detection was affected by the reflection/transmission produced in the concrete/inclusion/ concrete interface and the diameter-wavelength relation.

Finally, the experimental results showed that using a high-performance automatic ultrasonic inspection system the tomographic images are very useful for evaluating the internal inclusions of concrete structures. It was shown that the attenuation tomographic images allow to locate and to estimate the size of the inclusion; however, it was not possible to determine the type of embedded material. The results presented here shown that knowing a priori the internal structure of the specimen, as for example the dimensions, materials and theoretical positions of the reinforcements, as well as other embedded structures, water pipes, electric pipes, tendons, etc. is possible to detect any variation in the structure and any process that produces a change in the iron-concrete interface by tomographic imaging. For example, if we know that the reinforcement of a structure is of a certain diameter and by ultrasonic tomography we measure that its diameter is larger, the most probable conclusion is that microcracking of the concrete surrounding the reinforcement has occurred, either due to the corrosion or some other deterioration process. As it has been seen in this work, the voids are oversized and therefore the extension of the damage will be amplified in the image. Other possibility could be that a reinforcement of a larger diameter has been used but this change could be readily detectable by the uniformity of the reinforcement image.

## Figures and Tables

**Table 1:** Material and size of the embedded inclusion in circular scenarios of concrete.

Materials	Inclusion 1 (I-1, Ø, mm)	Inclusion 2 (I-2, Ø, mm)	Inclusion 3 (I-3, Ø, mm)
PVC ring	20	25	32
Aluminium	16	22	30
Steel	16	19.5	28

**Table 2:** Properties of materials used in the numerical simulation

Material	Density (kg/m^3^)	Longitudinal Velocity (m/s)	Transversal Velocity (m/s)
Cement paste	2300	4099.31	2295.55
Aggregates	2600	5721.25	3282.24
Air	1.24	340	
Water	1000	1480	
Aluminium	2720	6320	3772
Steel	7800	5850	3220
PVC	1400	2381	1045

**Table 3:** Description diameter and size of the embedded inclusion in concrete.

Materials	Inclusion 1 (I-1)	Inclusion 2 (I-2)	Inclusion 3 (I-3)
PVC tube	Ø 20 mm H = 70 mm	Ø 25 mm H = 120 mm	Ø 32 mm H = 170 mm
Aluminium bar	Ø 16 mm H = 70 mm	Ø 22 mm H = 120 mm	Ø 30 mm H = 170 mm
Steel bar	Ø 16 mm H = 70 mm	Ø 19.5 mm H = 120 mm	Ø 28 mm H = 170 mm

**Table 4:** Concrete mix proportion.

Material	Cement (kg/m^3^)	Sand (kg/m^3^)	Aggregates (kg/m^3^)	Water (kg/m^3^)	Superplasticizers (% of Cement Weight)
Amount	433	983	1000	177.53	0.01

**Table 5:** Detection and estimated dimensions (area and diameter).

		Real Dimensions			Detectability (%)	Estimated dimensions					
		*A* (mm^2^)	*D* (mm)	*L* (mm)		$\hat{A}$ (mm^2^)	$\sigma_{\hat{A}}$ (mm^2^)	$\varepsilon_{A}$ (%)	$\bar{D}$ (mm)	$\sigma_{\bar{D}}$ (mm)	$\varepsilon_{D}$ (mm)
PVC	I-3	804	32	170	98	1049	230	30	36	4	4
	I-2	491	25	120	83	508	192	3	25	5	0
	I-1	314	20	70	26	392	52	25	22	2	2
Al	I-3	707	30	170	60	560	154	−21	26	4	−4
	I-2	380	22	120	33	297	134	−22	19	5	−3
	I-1	201	16	70	14	149	117	−26	13	6	−3
Steel	I-3	616	28	170	98	627	141	2	28	3	0
	I-2	299	19.5	120	83	301	112	1	19	4	0
	I-1	201	16	70	23	193	165	−4	14	8	−2
